# The Role of the Choroid in Stargardt Disease

**DOI:** 10.3390/ijms23147607

**Published:** 2022-07-09

**Authors:** Solmaz Abdolrahimzadeh, Martina Formisano, Mariachiara Di Pippo, Manuel Lodesani, Andrew John Lotery

**Affiliations:** 1 Ophthalmology Unit, Neurosciences, Mental Health, and Sense Organs (NESMOS) Department, Faculty of Medicine and Psychology, University of Rome Sapienza, 00185 Rome, Italy; mariachiara.dipippo@uniroma1.it (M.D.P.); manuel.lodesani@uniroma1.it (M.L.); 2 St. Andrea Hospital, Via di Grottarossa 1035/1039, 00189 Rome, Italy; 3 St. Maria della Misericordia Hospital, Viale Tre Martiri 140, 45100 Rovigo, Italy; martinaformi@gmail.com; 4 Clinical and Experimental Sciences, Faculty of Medicine, University of Southampton, Southampton SO16 6YD, UK; a.j.lotery@soton.ac.uk

**Keywords:** Stargardt disease, retinal dystrophy, spectral domain optical coherence tomography, choroidal vascularity index, choriocapillaris vascular density, choroidal thickness, optical coherence tomography angiography, enhanced depth imaging, fluorescein angiography, indocyanine green angiography

## Abstract

Stargardt disease is the commonest juvenile macular dystrophy. It is caused by genetic mutations in the ABCA4 gene. Diagnosis is not always straightforward, and various phenocopies exist. Late-onset disease can be misdiagnosed with age-related macular disease. A correct diagnosis is particularly critical because of emergent gene therapies. Stargardt disease is known to affect retinal pigment epithelium and photoreceptors. Many studies have also highlighted the importance of the choroid in the diagnosis, pathophysiology, and progression of the disease. The choroid is in an integral relationship with the retinal pigment epithelium and photoreceptors, and its possible involvement during the disease should be considered. The purpose of this review is to analyze the current diagnostic tools for choroidal evaluation and the extrapolation of useful data for ophthalmologists and researchers studying the disease.

## 1. Introduction

Stargardt disease (STGD) is the most common juvenile-onset macular dystrophy [1]. It is caused by mutation of the ABCA4 gene, which results in an error in the processing and transportation of all-trans retinaldehyde, from the photoreceptor visual cycle, leading to the formation of N-retinylidene-N-retinyl-ethanolamine (A2E) and the storage of lipofuscin within the photoreceptors’ outer segments and the retinal pigment epithelium (RPE) [2]. STGD typically presents with progressive bilateral loss of central vision. It is classically characterized by a “beaten-bronze” or bull’s eye appearance of the macula and the presence of subretinal lipofuscin flecks [3]. Though it typically affects young patients, STGD may present later in adulthood and can be misdiagnosed as age-related macular degeneration (AMD) [4].

There are pathogenic variants in the ABCA4 gene, referred to as ABCA4opathy, that show different phenotypes that differ from typical STGD. The classical phenotype is due to biallelic pathogenic variants in the ABCA4 gene. Phenocopies can be caused by heterozygous pathogenic variant in PROM1 or ELOVL4, which can have a clinical profile indistinguishable from classic STGD1 [5,6]. Other types of macular dystrophy can also mimic STGD. The term “Stargardt” is often used to describe a wide variety of maculopathies with and without lipofuscin deposits. However, precise diagnosis, aided by genetic testing, is warranted for appropriate genetic counselling, prognostic information, and clinical trials due to the fact of emergent gene therapies [6].

An important question relates to the role of the choroid in STGD and whether alterations in the choroid affect the diagnosis and management of patients. Lipofuscin accumulates in the RPE nearly seven times more than normal in STGD [7]. This can alter the relationship between the RPE and the underlying choroid, which are interdependent. The choroid provides vascular support to the RPE which, in turn, maintains the choriocapillaris by secreting vascular endothelial growth factor isomers. Animal studies have shown secondary choriocapillaris atrophy after RPE atrophy, apparently because of a lack of RPE-specific vascular endothelial growth factor isomers [8]. Histopathological examination has also shown the loss of RPE, overlying photoreceptors, and choroidal circulation, in particular the choriocapillaris [9,10]. Interestingly, Mucciolo et al. recently described choroidal caverns using structural spectral domain optical coherence tomography (SDOCT) in 23 of 172 eyes of patients with STGD. These choroidal alterations were described as hyporeflective cavities with a hyperreflective rim and a nonhomogenous hyperreflective tail in the Sattler and Haller layers. Although the origin of these cavities is still not clear, given their location beneath areas of RPE atrophy in advanced STGD, the authors postulated that they could be related to RPE impairment and consequent choroidal sclerosis [2,11].

## 2. Fundus Autofluorescence and Near Infrared Autofluorescence

**Figure 1:**
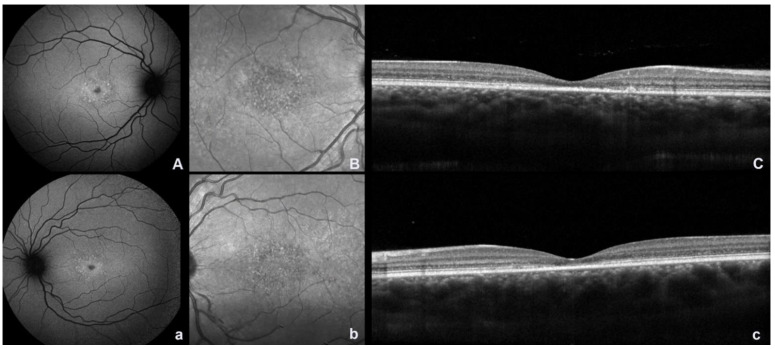
Fundus autofluorescence (FAF), near-infrared reflectance (NIR), and spectral domain optical coherence (SDOCT) images in a patient with bilateral molecularly confirmed ABCA4 Stargardt disease in the early stage. (**A**,**a**) FAF image showing a small, nascent region of macular hypoautofluorescence with a surrounding ring of hyperautofluorescence; (**B**,**b**) NIR image showing an area of dotted hyper–hyporeflectivity of the macula; (**C**,**c**) SDOCT scan showing foveal alterations of the outer retinal layers (uppercase and lowercase letters indicate the right and left eye, respectively).

**Figure 2:**
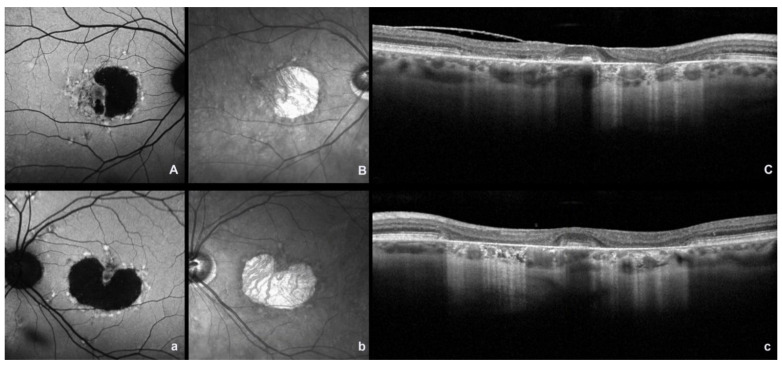
Fundus autofluorescence (FAF), near-infrared reflectance (NIR), and spectral domain optical coherence (SDOCT) images in a patient with bilateral molecularly confirmed ABCA4 Stargardt disease in an advanced stage. (**A**,**a**) FAF image showing a diffuse area of macular hypoautofluorescence with surrounding hyperautofluorescenct flecks; (**B**,**b**) NIR image showing a central macular hyporeflective area (atrophy area) surrounded by dotted hyperreflectivity (flecks); (**C**,**c**) SDOCT scan showing the absence of the outer retinal layer with diffuse backscattering (uppercase and lowercase letters indicate the right and left eye, respectively).

Multimodal imaging techniques are essential for making a diagnosis and confirming the clinical suspicion of disease. Fundus autofluorescence (FAF) is a noninvasive technique that provides information on the metabolic status of the RPE [3]. Short-wavelength fundus autofluorescence (SW-AF) demonstrates RPE atrophy as areas of hypoautofluorescence. The typical picture is a small, nascent region of macular hypoautofluorescence with a surrounding ring of hyperautofluorescence, given by lipofuscin accumulation, resembling a bull’s eye phenotype (Figure 1 and Figure 2). The signal of SW-AF is derived from lipofuscin in the RPE. The near-infrared autofluorescence (NIR-AF) signal originates from melanin in the RPE and the choroid. It has been thought that NIR-AF can better delineate the degree of RPE atrophy [12] (Figure 1 and Figure 2). The full-field electroretinogram is usually normal or minimally abnormal, whereas multifocal electroretinogram (mf-ERG) may show a more demonstrable reduction of central retina responses [6].

## 3. Fluorescein Angiography and Indocyanine Green Angiography

Many studies have focused on the choroidal alterations using multimodal imaging. Fluorescein angiography (FA) and indocyanine green angiography (ICGA) are the gold standard in the study of the eye’s vascular circulation. It has been demonstrated that a high rate of patients with STGD show masking of choroidal fluorescence on intravenous FA, also known as a dark choroid. This represents the earliest stage of the disease and is thought to be related to abnormal blue–green absorbing material rather than to a nonfilling of the choroid [13]. Another finding is choroidal silence, consisting of hypofluorescence in ICGA late frames, referred to as the marked atrophy of the choriocapillaris [10]. The peripheral flecks typically found in the fundus of patients with STGD appear as hypofluorescent at ICGA with surrounding hyperfluorescent edges. The areas of macular atrophy allow visualization of the choroidal hyperfluorescence [13].

Schwoerer et al. described the appearance of hypofluorescent curvilinear areas on ICGA forming a reticular pattern like the polygonal shape of the watershed zones between terminal choroidal arterioles. The authors attributed this aspect to possible choriocapillaris defects secondary to RPE cell damage. The patchy areas of hyperfluorescence seen on FA, indicating partially lysed RPE cells, corresponded in location to the hypofluorescent lesions of ICGA, although they did not correspond to the flecks seen on ophthalmoscopy [13]. The authors postulated that RPE cells could face some difficulty in meeting their metabolic needs. This correlated with the watershed zone between terminal choroidal arterioles, with worsening of intracellular lipofuscin elimination and further overload of the cells. They speculated that this could explain the ophthalmoscopic appearance and distribution of the flecks and the ICGA picture [13].

Giani et al. compared ICGA aspects in STGD and AMD to relate the areas of retinal atrophy to choroidal status and found that hypocyanescence with ICGA of areas of atrophy had a peculiar aspect in STGD with respect to atrophic AMD. In STGD, there was hypocyanescence at ICGA with a relative intact choroid and a lack of leakage on FA, suggesting possible direct damage to the choriocapillaris [14]. The identification of choriocapillaris (CC) atrophy represents a possible diagnostic tool, especially for patients with late-onset of the disease [3].

## 4. Optical Coherence Tomography Angiography and Spectral Domain Optical Coherence Tomography

FA and ICGA can provide indirect findings but cannot show the deep microvascular capillary complex. Optical coherence tomography angiography (OCTA) is a noninvasive tool that permits the study of the superficial and deep retinal vascular plexi and the CC in detail [3]. Individual CC vessels are not visualized but rather appear as homogeneous, with an area of brightness representing blood flow [12]. CC loss in STGD has been shown in many OCTA studies [3,7,15,16,17,18]. Choriocapillaris vascular density (CCVD) is calculated as the percentage of the area occupied by CC blood vessels, evaluated for an entire enface angiogram, and Battaglia Parodi et al. showed that CCVD was worse in STGD eyes with chorioretinal atrophy [17]. Both Pellegrini et al. and Muller et al. [3,18] found that the CC flow signal was visibly diminished within areas of RPE atrophy in STGD but not outside areas of RPE atrophy. Alabduljalil et al. reported that CCVD within areas of isolated RPE atrophy in STGD patients was significantly lower than in normal eyes, but this aspect was also present in areas of isolated inner segment/outer segment (IS/OS) loss. Moreover, the CCVD in areas of combined RPE and IS/OS attenuation tended to be even more altered, supporting the theory that the degeneration of both layers is synergistically associated with CC attenuation in STGD. The extent of total CCVD attenuation is strongly correlated with the magnitude of RPE and photoreceptor degeneration [19]. Alabduljalil et al. also demonstrated that the total CCVD was more strongly correlated than intralesional CCVD to the size of the degenerative lesion. This suggests that extralesional CCVD contributes to the level of total CCVD attenuation in STGD. The authors postulated that these findings were indirectly supported by microperimetry studies by Strauss et al. [20], who reported extralesional areas of decreased cone- and rod-dependent sensitivity. These results suggest that relative photoreceptor dysfunction with mild CC attenuation may precede lesion expansion in STGD.

Some studies reported correlation of IS/OS loss, RPE atrophy, or CCVD with age and/or best corrected visual acuity (BCVA). Ratra et al. and Mastropasqua et al. showed that CC vascular index and CCVD correlated with decreasing BCVA and parafoveal macular thickness, respectively [7,16]. Alabduljalil et al. reported a trend towards moderate correlation between IS/OS loss, RPE atrophy, and CCVD attenuation with age or BCVA [19].

Jauregui et al. studied the progression of CC impairment along with RPE atrophy in 55 patients with a clinical and molecular diagnosis of STGD. Based on their results, the authors divided the patients into three groups. In group 1, the patients had normal CC. In group 2, there was bright macular CC which could have misleadingly been attributed to increased blood flow but was actually due to the presence of an artifact because of the disappearance of the overlying RPE. This was confirmed by corresponding atrophy on SW-AF. In group 3, there was vascular rarefaction and incomplete CC atrophy. The authors found that group 1 patients corresponded to a G1961E allele. This allele is associated with milder disease severity, as also reported in other studies. They also found a dark halo of the lesion perimetry, probably corresponding to a shadow cast by lipofuscin accumulation in the RPE overlying the CC. These results demonstrate the advantage of using OCTA imaging in monitoring disease, and the authors concluded that as the RPE becomes atrophic, the CC disappears as a downstream effect [12].

## 5. Hyperreflective Foci

A possible pathologic marker in STGD is the presence of hyperreflective foci (HRF). HRF are solitary, small (<30 µm), medium-level hyperreflective retinal foci that may represent aggregates of activated microglial cells, in vivo SDOCT biomarkers of retinal inflammation. They were recently reported to estimate the amount of inflammation in major degenerative retinal [21] and chorioretinal diseases including AMD, retinal vein occlusion, and diabetic retinopathy [22,23,24]. Intraretinal and choroidal HRF were described in patients with early retinitis pigmentosa (RP) [25]. A study performed with SDOCT analysis demonstrated HRF in the choroid, mostly in the Bruch’s membrane/RPE complex in the CC and in Sattler’s layer [26], and choroidal HRF were associated with retinal atrophy, disease duration, and visual function. Although the presence of HRF were detected in both RP and SGDS the direct significance to pathology is unknown, particularly regarding visual function. It was, however, postulated that choroidal HRF may be associated with CC dysfunction [27].

## 6. Choroidal Thickness

Changes in choroidal thickness (CT) and morphology have also been the object of debate in patients with STGD. Enhanced depth imaging (EDI) SDOCT permits analysis of the detailed structure of the choroid. CT and the choroidal vascularity index (CVI) can be assessed. However, results are discordant, as some authors reported a significant reduction in CT in STGD [28,29,30] when compared with healthy controls, while other authors did not report significant differences [31,32,33]. The largest study on CT was conducted by Sabbaghi et al. on 264 eyes with RP, 76 with STGD, and 54 with Usher syndrome [33]. However, only 19.5% of the diagnosis was genetically confirmed. In this study, the mean CT was significantly lower in patients with RP and Usher syndrome when compared with healthy controls and patients with STGD and cone–rod dystrophy. There was a statistically significant correlation between thinner subfoveal CT and longer duration of ocular symptoms in STGD. A negative correlation was observed between the subfoveal CT and BCVA and between subfoveal CT and disease duration in the inherited retinal dystrophies. However, no correlation was observed between central macular thickness and CT [33]. Other studies report a correlation between CT and BCVA and between CT and the duration of ocular symptoms in inherited retinal dystrophies including RP and STGD [28,29,31,34], but other authors did not find this correlation [30,31,32,35,36,37]. Sabbaghi et al. suggested that this may be related to low sample sizes and shorter durations of symptoms [33]. Vural et al. found decreased subfoveal and parafoveal CT in patients with STGD and correlated this with lower BCVA, inner retinal thickness, and paracentral mf-ERG responses. Adhi et al., in accordance with Vural et al., found that the mean CT thickness in healthy eyes was lower in the nasal sector, greater in the subfoveal region, and thinner temporally, as also reported by Manjunath et al. [28,29,38]. Adhi et al. also found that that the maximum CT was not subfoveal in 79% of patients, and focal thinning was observed in 51%, with maximum thinning in the nasal region in 30% of patients with STGD. Moreover, the average subfoveal CT and the mean thickness of the large choroid vessels layer (LCVL) were significantly reduced in the eyes with STGD. The average ratio between LCVL and total CT was significantly reduced in eyes with STGD compared to healthy eyes. Substantially, this study also analyzed choroidal morphology in eyes with STGD, compared to healthy eyes and reported a correlation between the reduction of thickness of the LCVL and reduction in BCVA in patients. Adhi et al. suggested that the choroid is irregularly shaped or “S”-shaped (having an irregular concave/convex/concave shape with more than one inflection point) in over two-thirds of eyes with STGD [28]. An exaggerated nasal choroidal thinning was also shown in a previous study in eyes with RP [39]. In addition, in the eyes with STGD, the mean thickness of the subfoveal choroid was positively correlated with central retinal thickness, indicating that total CT decreased with central retinal thickness thinning. However, the average thickness of the LCVL and the ratio of this layer to the thickness of the choroidal layer had little correlation with central retinal thickness (*p* = 0.45). Another noteworthy fact is that the attenuation or preservation of the LCVL in the eyes with STGD was not associated with the presence or absence of atrophy of the RPE and external retinal atrophy. However, two limitations of the study by Adhi et al. were the absence of molecular diagnosis of STGD and that the SDOCT images were not conducted with EDI [28].

Ratra et al., in 2018, described a thinner choroid in 26 patients with STGD. Statistically significant values were reported for the 1 mm central and the superior and temporal sectors of the 3 mm ring [30]. In STGD, the total CT and thickness of the small choroidal vessel layer (SCVL) were reduced, but the LCVL was unaffected or increased in thickness. The authors related CC atrophy to the “dark choroid” appearance on FA and ICGA and suggested that increased thickness of the LCVL may be a compensatory response to the atrophy of the SCVL in order to maintain the blood supply [30]. Ratra et al. suggested that RPE atrophy is more likely to cause thinning of the CC due to the fact of their proximity and symbiotic relationship. They also did not find any correlation between BCVA and total CT, but there was a correlation between the lesion size and BCVA worsening [30]. The patients studied by Adhi et al. were at a more advanced stage of disease with advanced choroidal damage, and these authors observed attenuation of the LCVL with correlation to visual acuity. Total CT was almost stable inside the lesion and at the edge of the lesion while showing a slight decrease outside the lesion on the nasal side. The SCVL showed a gradual decrease from the outside to the inside of the lesion similar to the pattern of retinal layer thickness, while the LCVL showed a gradual increase from the outside of the lesion towards the inside. However, the differences were not statistically significant [28,30]. Muller et al. reported no significant difference in subfoveal CT of patients carrying the common ABCA4 mutation (p.Gly1961Glu), but these patients had no evidence of RPE atrophy and had normal scotopic and photopic responses on ERG. Nevertheless, in the presence of RPE atrophy, the choroid was thinner, and the inner choroid was more affected [40]. One study reported mildly increased CT compared to normal controls, but the study was driven by a single case report with markedly thicker choroid [41]. Table 1 summarizes the studies where CT was evaluated in STGD (Table 1).

## 7. Choroidal Vascularity Index

CVI is a parameter calculated using the ratio of luminal area/total choroidal area (TCA). A polygon tool is used to select the TCA from a foveal SDOCT scan. A color threshold tool is then applied to the TCA, and the dark pixels represent the luminal area, whereas the light pixels the stromal or interstitial area [7]. In a large cross-sectional study involving healthy subjects, CVI but not CT was reported to be independent from systemic and ocular factors including age, systemic blood pressure, axial length, and intraocular pressure. This made it a more reliable disease biomarker with less influence by confounding factors [42]. Moreover, CVI encompasses changes in both the vascular and stromal component of the choroid, adding structural information compared to CT [7].

Ratra et al. studied choroidal vascularity in 39 patients with STGD and analyzed the subfoveal choroidal thickness (SFCT) and the CVI. The average CVI of STGD patients was lower than in the control group with statistically significant values. A decrease in the CVI was also correlated with a decrease in visual function. The SFCT was lower in the study group than in the controls, but these differences were not statistically significant. The authors concluded that CVI could be a more robust and sensitive tool compared with SFCT evaluation for choroidal changes in STGD. Moreover, a decrease in the luminal area but with a compensatory increase in the stromal area would lead to a decrease in CVI but not a decrease in SFCT. The authors concluded that CVI correlated with worsening BCVA. This could possibly be used as a biomarker to monitor progression of disease and worsening of vision [7].

Wei et al. studied CVI in patients with various retinal dystrophies (17 patients with RP, 4 with STGD, and 3 with cone–rod dystrophy) and reported that the mean CVI in eyes with retinal dystrophy was significantly lower than in healthy controls (52.9% vs. 70.3%). All types of retinal dystrophy in this study were associated with lower CVI after adjustment for age, sex, visual acuity (VA), and duration of symptoms. They deduced that choroidal ischemia may be implicated due to the progression and the pathogenesis of the disease [43].

## 8. Conclusions

The results of the present review contribute to the definition of STGD as a condition where there is an impairment of the choroid, both in terms of thickness and alteration of its vascular layers. Even though not all of the results from the various studies were in agreement or reached statistical significance, substantial evidence does exist. The CVI emerges as a noninvasive biomarker to distinguish retinal dystrophies from healthy controls [43]. Indeed, CVI is useful in the evaluation of the choroidal structure, because it examines changes in both the vascular and stromal components. This provides unique and additional structural information compared to the commonly reported CT. In patients with a clinical diagnosis of STGD, CVI appears to be a more sensitive biomarker in detecting choroidal changes than CT [7,14,28]. In addition, a significant difference in CVI, between patients with STGD compared to normal subjects, leads to the conclusion that in addition to the retina and the RPE, the choroidal layer is also involved, suggesting that choroidal angiopathy could be part of the pathogenesis of STGD [30]. Interestingly, with the same duration of the ocular symptoms, a generalized choroidal thinning was observed in RP and Usher syndrome but not in STGD and cone dystrophy. This suggests different pathophysiological and blood flow mechanisms [33].

Mild CC reduction has been observed in areas with relatively mild photoreceptor dysfunction. Therefore, this does not clearly delineate whether the CC or photoreceptors are affected first but suggests their interdependence. This implies it may be possible to predict functional damage by measurement of CC thickness [19]. Further studies of the choroid are also warranted to evaluate the impact of ABCA4 gene therapy on the choroid and its blood flow. In conclusion, the results of this review do not unambiguously define assessment criteria for STGD; however, it is clear that the evaluation of the choroid is valuable in assessing prognosis. EDI-SDOCT and OCTA are useful tools in the diagnosis and follow-up of STGD. They permit calculation and assessment of CT and, more importantly, the CVI. A limitation of the OCTA examination may be that patients with STGD often present with eccentric fixation, and this makes it difficult to perform the OCTA examination. Furthermore, the data provided by this innovative technique should be compared to microperimetry, visual acuity, SDOCT and, where possible, to the mf-ERG.

To further assess the value of SDOCT and OCTA, it would be valuable to include these imaging modalities routinely along with FAF when monitoring STGD patients. This should lead to better prognostic information for patients and a more refined genotype–phenotype correlations.

## Figures and Tables

**Table 1:** Clinical characteristics of studies analyzing choroidal thickness and vascularity alterations in patients with Stargardt disease.

Study	Study Design	Number of Patients (Number of Eyes) with Stargardt Disease/Controls	CT Alterations	CVI Alterations	Correlations between CT and BCVA
Yeoh et al., 2010 [35]	Prospective observational case series	Cases: 5	2 pts: no 3 pts: choroid thinning	-	No association between choroidal thinning and visual acuity
Sabbaghi et al., 2020 [33]	Comparative study	Cases: 38 (76) Controls: 56 (109)	No differences in mean total and SFCT	-	Inverse correlation of SFCT with BCVA
Ratra, Tan et al., 2018 [7]	Retrospective cohort study	Cases: 39 Controls: 25	No differences in SFCT	Decreased CVI in patients with Stargardt disease	Negative association between visual acuity and CVI Positive association between visual acuity and SFCT
Chhabblani et al., 2015 [32]	Retrospective study	Cases: 9 (18)	No choroidal thinning	-	No significant correlation between subfoveal CT and BCVA
Adhi et al., 2015 [28]	Cross-sectional retrospective review	Cases: 28 (53) Controls: 30 (30)	No differences in mean total and SFCT	-	No correlation between CT and BCVA
Ratra, Jaishankar et al., 2018 [30]	Case-control study	Cases: 26 (52) Controls: 26 (52)	Total CT decreased in cases	-	No correlation between CT and BCVA BCVA worsened with increasing lesion size and wider extent of flecks

BCVA: best corrected visual acuity; CT: choroidal thickness; CVI: choroidal vascularity index; SFCT: subfoveal choroidal thickness.

## Data Availability

Not applicable.
